# Recent Research on Hybrid Hydrogels for Infection Treatment and Bone Repair

**DOI:** 10.3390/gels8050306

**Published:** 2022-05-16

**Authors:** Mengjiao Cao, Chengcheng Liu, Mengxin Li, Xu Zhang, Li Peng, Lijia Liu, Jinfeng Liao, Jing Yang

**Affiliations:** 1 State Key Laboratory of Oral Diseases, National Clinical Research Center for Oral Diseases, Department of Cariology and Endodontics, West China Hospital of Stomatology, Sichuan University, Chengdu 610041, China; caomengjiao@stu.scu.edu.cn (M.C.); limengxin0103@stu.scu.edu.cn (M.L.); 2017151642186@stu.scu.edu.cn (L.L.); 2 State Key Laboratory of Oral Diseases, National Clinical Research Center for Oral Diseases, Department of Periodontics, West China Hospital of Stomatology, Sichuan University, Chengdu 610041, China; liuchengcheng@scu.edu.cn; 3 State Key Laboratory of Oral Diseases, National Clinical Research Centre for Oral Diseases, West China Hospital of Stomatology, Sichuan University, Chengdu 610041, China; zx2020224030006@163.com; 4 Key Laboratory of Bio-Resource and Eco-Environment of Ministry of Education, College of Life Sciences, Sichuan University, Chengdu 610065, China; pengli1@stu.scu.edu.cn

**Keywords:** hydrogels, antibacterial agents, infection control, bone repair

## Abstract

The repair of infected bone defects (IBDs) is still a great challenge in clinic. A successful treatment for IBDs should simultaneously resolve both infection control and bone defect repair. Hydrogels are water-swollen hydrophilic materials that maintain a distinct three-dimensional structure, helping load various antibacterial drugs and biomolecules. Hybrid hydrogels may potentially possess antibacterial ability and osteogenic activity. This review summarizes the recent progress of different kinds of antibacterial agents (including inorganic, organic, and natural) encapsulated in hydrogels. Several representative hydrogels of each category and their antibacterial mechanism and effect on bone repair are presented. Moreover, the advantages and disadvantages of antibacterial agent hybrid hydrogels are discussed. The challenge and future research directions are further prospected.

## 1. Introduction

With the advancement of society, the occurrence of high-energy injury events and the use of internal implants increased, as did the number of trauma and postoperative bone infection patients [1]. Each year, over 2 million bone transplants are applied nationwide [2]. Bone tissue has a limited capacity for regeneration and healing. For complex fractures and bone defects, early external intervention is frequently needed for successful recovery [3]. Generally speaking, a “critical-sized” defect is one that does not receive adequate blood supply for the callous formation and does not recover spontaneously after surgical stabilization, requiring subsequent intervention [3,4]. Critical-sized bone defects, which are typically associated with high-energy injuries or pathological fractures, remain to be a substantial therapeutic problem and necessitate bone transplantation. The defects might vary in severity depending on the site of the damage [5].

An acute and well-controlled inflammatory response is elicited and beneficial to healing when a bone injury occurs. Once the response is inhibited, dysregulated, or becomes chronic, it could be harmful to the healing process [6,7,8]. Inflammation is a critical physiological activity for pathogen elimination and tissue homeostasis preservation. Infected bone defects (IBDs) are chronic diseases with a complex pathology that typically lasts long and has an uncertain prognosis [9]. The healing time varies affected by the location and size of the defects, as well as the severity of the infection [10,11]. IBDs are frequently caused by a combination of acute high-energy injuries and contamination. These types of acute bone infections can occasionally lead to osteomyelitis and chronic infection. Opening fractures, soft tissue or bone tissue loss, infection following internal fixation, and a bone tumor are common causes [11]. Acute bone infections are typically treated with routine systemic antibiotics. Chronic infections and osteomyelitis often necessitate surgical debridement of necrotic tissues in combination with local antibiotic therapy [12].

Efficient elimination of inflammatory stimulants and the release of anti-inflammatory and reparative cytokines are required to treat infected diseases and restore tissue homeostasis [13]. However, the sequence of events can be changed by the presence of a pro-inflammatory stimulus, and the condition may turn to chronic inflammation. Immune cells, particularly macrophages, are important in regulating inflammation. Research on the interconnection between the immune system and bone metabolism led to the term “osteoimmunology” being coined to describe this new field [14]. The presence of both hematopoietic stem cells (HSCs) and mesenchymal stem cells (MSCs) in bone marrow emphasizes the strong connection between these two systems [15]. Bone-resorbing osteoclasts and immunomodulatory macrophages originate from HSCs, and bone-forming osteoblasts develop from MSCs [16]. Because of the shared origin of cytokines, receptors, signaling molecules, and transcription factors, osteoblasts and bone-resorbing osteoclasts of a monocyte/macrophage cell regulate one another [17,18].

Because of bacterial colonization and osteonecrosis, clinical treatment of IBDs has always been complex [19,20]. Surgical treatment of the infected bone frequently results in significant disabling defects. The implantation of bone grafting materials and antibiotic therapy are common treatment modalities for IBDs in clinic [21]. The presence of bacteria in infected bone and surrounding tissues can cause the release of inflammatory and tissue destructive mediators, interfering with osteogenesis [22]. One of the most difficult challenges in modern orthopedics is to eliminate bacterial infection and provide a biocompatible microenvironment for bone repair in bone defects. Because of the inadequate local blood supply, antibiotics in high concentrations are needed in the area of infection. However, conventional routes of drug administration are challenging to achieve excellent antibiotic effects and exacerbate serious side effects [23].

Bone grafts used to treat IBDs should act as osteoinductive bone substitutes and antimicrobial carriers [12]. Autologous bone, also known as autograft, is still regarded as the clinical “gold standard” for bone repair. However, there are several limitations to autogenous grafting associated with the harvesting process. The shortcomings include morbidity of the donor site, increased blood loss, and longer operating times [24]. Furthermore, the allograft is a limited supply of autologous bone substitutes because of the high expenses and dangers of viral transmission [24,25,26]. Fortunately, bone substitutes or synthetic grafts are intended to overcome the drawbacks of autologous and allogeneic bone grafts. When used to restore contaminated bone tissue, bone grafts should ideally inhibit local bacterial growth. Simultaneously, it should stimulate cellular infiltration and immunomodulatory effects in host reparative cells [27,28].

Fabrication of biomedical materials with good antimicrobial and osteogenic activities is critical for promoting the repair effects of bone substitutes on IBDs [29]. Several common materials have been extensively used in bone tissue engineering, including nanofibrous materials, coatings, and hydrogels [30]. In particular, hydrogels have porous network structures and good biocompatibility to mimic the extracellular matrix (ECM) [31]. As a distinct class of soft materials, hydrogels are composed of hydrophilic networks that can maintain moisture. Hydrogel is a suitable candidate to be used as carrier materials for cells or bone growth to facilitate growth factors released and can be easily loaded with antibacterial agents [32]. Hydrogels can be fabricated from polymer chains connected by physical interactions or chemical bonds, and varying crosslinking methods and degrees can easily control the degradation rate, porosity, or release profile [33]. Additionally, hydrogels can self-assemble with self-complementary amphiphilic peptides by gelation. Furthermore, they can be tailored to meet the optimum geometry for implantation or injection [34]. Hydrogels are appealing therapeutic delivery materials, presenting the great potential to encapsulate agents in the water-swollen network [35]. Additionally, some types of hydrogels have inherent antibacterial properties, such as chitosan (CS) and polyethyleneimine (PEI) [32,36,37]. So hydrogels are scaffolds that have been widely researched as a potential alternative material for antibacterial tissue engineering.

**Figure 1:**
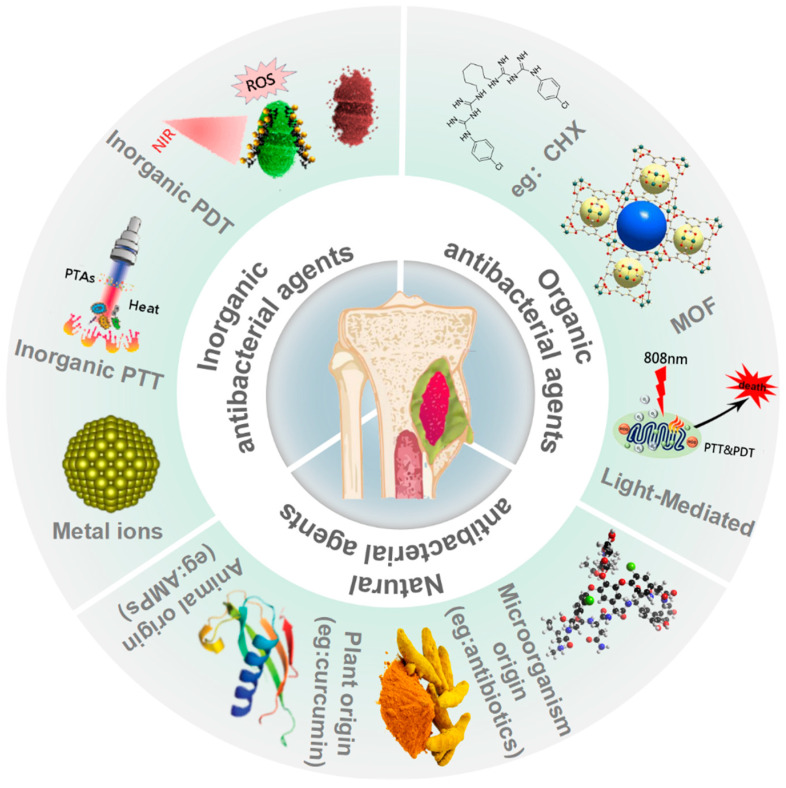
Antibacterial agents and their categories for infected bone defects.

Antibacterial agents can be classified into three types: inorganic antibacterial agents, organic antibacterial agents, and natural antibacterial agents based on their composition, source, and nature. Additionally, each type is sorted into different categories, as summarized in Figure 1.

Antibacterial agents administered systemically have a lot of drawbacks, such as low concentrations in the infected area and side effects. In comparison, local delivery of antimicrobial agents may offer appropriate antibacterial dosages [38]. Sustainable local delivery of antibacterial agents via a delivery carrier avoids many disadvantages of systemic side effects. Due to the excellent water content, great bioactivity, and convenience of drug-loading, hydrogels have been extensively researched as drug carriers for targeted delivery [39]. Antibacterial agents can be used in conjunction with hydrogels to slow down the kinetics of drug release and deliver the medication to the target site. Moreover, the hydrogels’ degradation rate can also be controlled, providing this material system the characteristics of a prolonged-release cycle and reducing administration dosage [40,41]. Therefore, hydrogels can encapsulate agents or agent-loaded nano-/microcarriers to provide sustained localized antimicrobial drug release for excellent antibacterial and bone repair performance [42]. This review will focus on recent research on antibacterial hydrogel systems in infected bone regeneration. The features of hybrid hydrogels in antibacterial mechanism and their effect on bone repair will be systemically presented.

## 2. Hybrid Hydrogels with Inorganic Antibacterial Agents for Infected Bone Repair

Inorganic antibacterial agents are classified based on their modes of action: metal ion elements (e.g., silver (Ag), gold (Au), copper (Cu), zinc (Zn)), and inorganic light-mediated antibacterial materials (e.g., reduced graphene oxide (rGO), carbon-based nanomaterial, titanium dioxide (TiO_2_), zinc oxide (ZnO) [43]. Light-mediated antibacterial activity can be achieved through photothermal therapy (PTT), photodynamic therapy (PDT), and sunlight-mediated antibacterial treatments [44]. There are few studies on sunlight-activated nanomaterials to date, so this review will focus on the PTT and PDT related inorganic light-mediated antibacterial agents.

### 2.1. Hydrogels with Metal Nanomaterials

**Figure 2:**
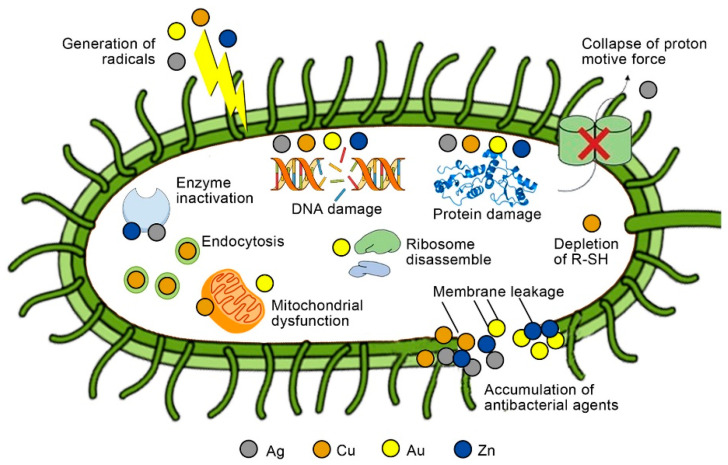
Possible antibacterial mechanisms for inorganic antibacterial agents of Ag, Cu, Au, and Zn. R-SH, sulfhydryls (Reprinted with permission from Ref. [50] Copyright 2021 Elsevier).

The antibacterial action of nanoparticles is achieved in a number of ways. Several factors, such as the released metal ions and the physicochemical characterization of nanoparticles, may lead to membrane disruption or cell wall penetration, which can contribute to nanoparticles’ antibacterial activity [45,46]. It has been shown that metallic nanoparticles (as in silver, gold, copper, and titanium) have significant antibacterial activity [47,48,49]. The mechanisms of inorganic antibacterial agents of several metal ions are illustrated in Figure 2.

Among the several metal nanomaterials applied in antibacterial therapy, silver nanoparticles (AgNPs) are the most extensively investigated antibacterial nanoagent with a broad antibacterial spectrum [51,52]. AgNPs are typically assumed to perform antibacterially by attaching to the cell wall and membrane, and then destroying the structures and biomolecules within the cell with AgNPs and silver ions [53,54,55]. At the same time, AgNPs can promote bone formation and accelerate the rehabilitation of injured tissues. Mahmood M et al. demonstrated that AgNPs could regulate many osteogenic genes related to bone growth [56]. Han et al. described a method to synthesize AgNPs-loaded hydrogels using gelatin (Gel) as a stabilizing agent in a simple way under sunlight, which improved the survivability and proliferation of osteoblasts on the hydrogels for bone fracture treatment [57].

Gold nanoparticles (GNPs) are also gaining immense attention since their antimicrobial activity has been reported [58]. After intracellular uptake, GNPs have been demonstrated to stimulate osteogenic differentiation and mineralization in cells [59,60]. For example, Zhang et al. prepared PEG-hydrogels with GNPs of 4 nm, 18 nm, and 45 nm in size. The results indicated that hydrogels containing GNPs of 45 nm could efficiently induce bone regeneration in vivo by increasing the osteogenic gene expression, mineralization, and alkaline phosphatase (ALP) activity [61]. In another case, Lee D et al. designed a hydrogel that tyramine (Ty) bound with the Gel backbone (Gel-Ty) containing GNPs attached to N-acetyl cysteine (NAC) (Gel-Ty/G-NAC) for effective bone regeneration [62]. Furthermore, GNPs can be utilized for PTT to treat tumors when exposed to near-infrared light [63]. In addition, copper nanoparticles show excellent antibacterial ability for both Gram-positive bacteria (GPB) and Gram-negative bacteria (GNB) [64]. For example, Dai Q et al. fabricated a unique 3D-printed Ty-modified Gel/silk fibroin (SF)/copper (Cu)-doped bioactive glass (BG) hydrogel [65]. The hydrogel with 1 wt% Cu-BG can effectively modulate osteogenesis and vascularization’s spatiotemporal coupling.

Like antibiotics, prolonged usage of AgNPs results in the development of multidrug-resistant microorganisms [66]. Unfortunately, inorganic nanoparticles are difficult to biodegrade in vivo. So the toxicity of inorganic nanoparticles should be reduced by surface modification.

### 2.2. Light-Mediated Inorganic Antibacterial Hydrogels

In comparison to traditional antibiotics, PTT would not induce bacterial resistance [67]. Aside from metal NPs, various photothermal agents (PTAs) have been successfully used in the antimicrobial field. PTAs can convert light into heat, resulting in rupture of the cell membrane, protein denaturation, and microbial death [68]. PTT has demonstrated significant promise in antibacterial and bone regeneration treatment due to the rapid development of different PTAs. The inorganic nanomaterials with PPT abilities include metal nanomaterials (Au, Pt), carbon-based nanomaterials (graphene, fullerene, rGO), black phosphorus (BP), and other metal oxide nanoparticles [44,69,70]. Unlike PTT, PDT generates reactive oxygen species (ROS) to generate cytotoxicity. Three elements are required for PDT: light, molecular oxygen, and photosensitizers (PSs). When the PSs are irradiated with light whose wavelength meets the PSs’ absorption, singlet oxygen (^1^O_2_), hydroxyl radicals, or oxygen-free radicals can be produced. These radicals can destroy cell membranes and DNA molecules [71].

Nanoparticles with photothermal and photodynamic ability have recently received much attention as a potential treatment for bacterial infections and bone healing. Geng et al. developed a multifunctional biodegradable gelatin/methacrylate anhydride (GelMA) hydrogel by controlling the surface charge and preventing the positive- and negative- charged carbon quantum dots (CQD)from aggregating [72]. They deposited positively charged carbon quantum dots (p-CQDs) on the surface of tungsten disulfide (WS_2_) nanosheets. Additionally, Geng et al. incorporated (p-CQDs)/WS_2_ with antimicrobial effects and negatively charged CQDs (n-CQDs) with bone induction ability in GelMA hydrogels. Not only can the hydrogels effectively kill multidrug-resistant bacteria (MDR), but they also considerably accelerate bone regeneration. Graphene, a typical carbon-based nanomaterial, has been extensively investigated for its ability to stimulate bone formation through interaction with osteoprogenitors and other skeletal progenitors. rGO is the product of treating graphene oxide (GO) under thermal, chemical, or UV [73]. In addition to improving mechanical properties, graphene family materials uniformly dispersed into polymers to produce materials can also promote cell proliferation and differentiation, hence facilitating bone regeneration [74]. Wang et al. fabricated the NIR light-responsive, rGO-loaded CS hydrogel films by electrodeposition [75]. The histological and radiological examination revealed that the films promoted bone regeneration in calvarial defect osteoporotic models. Li et al. developed hybrid hydrogels containing gelatin methacrylate, β-cyclodextrin-modified rGO, and acryloyl-β-cyclodextrin for infected skull defects [76]. These hydrogels exhibited ideal antibacterial photothermal properties, as well as unswelling and mechanical properties.

The difficult biodegradation of GO limits its biomedical applications, particularly in vivo [77]. Conversely, BP can degrade in aqueous conditions, generating harmless phosphates and phosphonates that promote biomineralization and regulate osteogenesis [78,79]. As a recently emerged 2D nanomaterial, BP has stimulated widespread research interest. For example, Miao et al. reported that the BP/Gel hydrogel could promote osteogenesis in vitro without osteoinductive factors. In the Sprague Dawley rat model, they also found considerable newborn cranial bone tissue growth [80].

**Figure 3:**
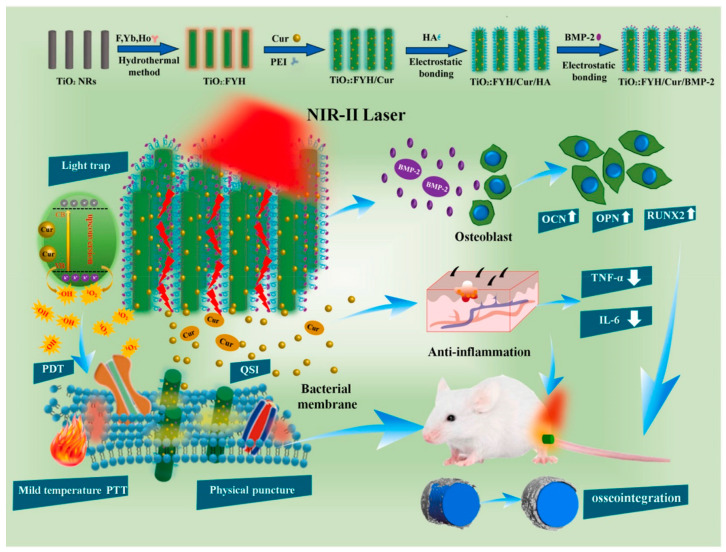
Schematic illustration of the crafting process of the TiO_2_: FYH/Cur/BMP-2 NRs on Ti implant towards biofilm elimination, anti-inflammation, and bone regeneration. OCN, osteocalcin; OPN, osteopontin; RUNX2, runt-related transcription factor 2; QSI, quorum-sensing inhibitors; TNF-α, tumor necrosis factor-α; IL-6, interleukin-6 (Reprinted with permission from Ref. [86]. Copyright 2021 Elsevier).

The human body is capable of withstanding high heat for a brief period of time, but normal cells in the surrounding area could be damaged [81,82]. The NIR light frequently employed for PTT therapy has a limited penetration depth [83]. In comparison to NIR-I light (650–1000 nm), the NIR-II window (1000–1700 nm) exhibits a greater penetration depth in tissue and lower energy attenuation [84,85]. Additionally, the combination of PDT and PTT can significantly enhance the antibacterial efficiency of phototherapy. As shown in Figure 3, Zhang et al. designed a NIR-II phototherapy system using ytterbium (Yb), erbium (Er), and holmium(Ho) co-doped TiO_2_ nanorods (TiO_2_ NRs) (TiO_2_:FYH)/curcumin (Cur)/hyaluronic acid (HA)/bone morphogenetic protein-2 (BMP-2) [86]. It had antibiofilm, anti-inflammatory, and osteogenic capabilities in vitro and in vivo. The temperature increased to 47 °C when the 1060 nm laser was used, which was higher by about 7.2 °C than that of the 808 nm laser in the rabbit femur. Furthermore, the system exhibited great antibiofilm capability in the rabbit femur when irradiated with a 1060 nm laser, while numerous microorganisms lived when irradiated with an 808 nm laser. Then, on a titanium bone implant, they constructed a NIR-II-triggered nano-platform made of Yb and Er-doped TiO_2_ nano-shovel (TiO_2_@UCN)/quercetin (Qr)/L-arginine (LA) [87]. When irradiated with a 1060 nm laser, the nanoplatform can eradicate biofilms on the titanium implants at 45 °C. Furthermore, the nano-platform enhanced revascularization and osteogenic differentiation, reduced inflammation, and promoted the generation of bone structures.

High temperatures and ^1^O_2_ from the phototherapy could easily destroy adjacent tissues, such as the periosteum and blood vessels [88]. PSs can also be developed to be activated by enzyme-mediated luminescence techniques in addition to external sources of excitation, allowing them to address depth constraints [89]. Developing near-infrared light-triggered nanomaterials with extremely prolonged luminescence lifetimes, allowing for continuous activation of PSs for phototherapy, may provide another way to avoid external light irradiation [70].

## 3. Hybrid Hydrogels with Organic Antibacterial Agents for Infected Bone Repair

Organic antibacterial agents including glutaraldehyde, quaternary ammonium salt compounds, and chlorhexidine (CHX), have been extensively studied [90,91,92]. Metal-organic frameworks (MOFs) are effective against bacteria. MOFs usually refer to composites with a network structure by the self-assembly of metal ions and organic ligands. In comparison to traditional bactericidal materials, MOFs exhibit larger specific surface areas, more adjustable pore structures, and controllable ion release rates. As a result, MOFs have a promising future in infected bone regeneration [93]. In addition to the inorganic photothermal materials mentioned above, organic photothermal agents have received much attention in recent years.

### 3.1. Hybrid Hydrogels with Organic Antibacterial Agents

Most inorganic antibacterial agents appear in the form of metal ions to kill GNB, whereas GPB are sensitive to organic antibacterial compounds via organelle modification and disruption of metabolic processes [50]. There are many organic antibacterial agents, such as CHX, organic acids, phenols, and quaternary ammonium compounds [94,95].

Quaternary ammonium salts (QAS) are important synthetic organic antimicrobials with a broad antimicrobial spectrum. QASs’ hydrophobic and ionic interactions with biological membranes damage microorganisms’ barriers [96,97]. For example, Lin et al. used quaternary ammonium chitosan (QTS) as a liquid phase in conjunction with calcium silicate (CaSi) powder to form cement [98]. When considering the osteogenic capacity, the antibacterial ability, and the setting time, the results revealed that CaSi cement with1% QTS might be a promising choice for bone regeneration.

Like QAS, CHX is commonly applied by healthcare personnel for general disinfection and hand hygiene [99]. CHX is a broad-spectrum antimicrobial material that inhibits the formation of biofilms and GPB/GNB growth, particularly against E. faecalis [100]. The antibacterial effect of CHX is mediated by the cation’s electrostatic interaction with the negatively charged portions of the bacterial surface, interfering with physiological activities and osmotic regulation in bacteria [101]. Xu L et al. developed a novel injectable hydrogel composed of nanohydroxyapatite particles and CHX (nHA/CHX) loaded in gellan gum (GG), which has the potential to enhance the repair of IBDs [102]. Bacteria counts were considerably lower in the surrounding bone tissue of rats treated with surgical debridement and GG/nHA/CHX transplantation than in the control group. Additionally, at 4 and 8 weeks, rats in the hydrogel group demonstrated considerably abundant new bone formation compared to the control group.

The antibacterial actions of the various organic antibacterial agents encompass a variety of distinct methods, including breaking down cell membranes or oxidizing the proteins and amino acids inside bacteria [103]. However, organic antimicrobials have some limitations in biodegradability, stability, and lifetimes [104]. For overcoming these problems, MOFs may be the solution.

### 3.2. Hybrid Hydrogels with Metal-Organic Frameworks

Due to the rapid rate of evolution of bacteria, the resistance of bacteria to many organic antimicrobial agents is increasing, which is an urgent problem in the healthcare system [105]. MOFs have attracted substantial attention recently as an innovative and fast-evolving group of organic-inorganic hybrid materials [106]. The majority of MOFs display antimicrobial properties by decomposing metal-ligand bonds and releasing ligands or metal ions into the bacteria. Additionally, they can be used as medication carriers through the adsorption or binding of medicines to their surfaces [107,108]. Various metal ions have been shown to have different effects on osteogenesis and bone mineralization, and their mechanisms of action have also been investigated. As a result, it was established that MOFs enhance osteogenic differentiation in vitro. In vivo studies were less common, which means that the application of MOFs for orthopaedic implants is just starting to be investigated [109].

**Figure 4:**
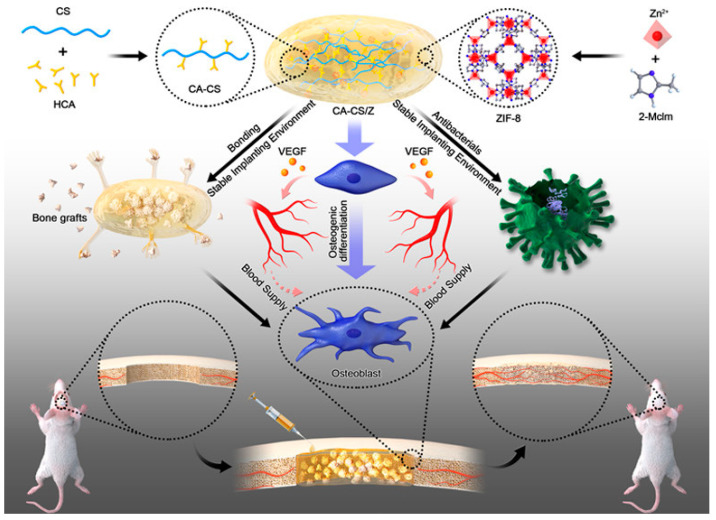
Scheme of the fabrication of CA-CS/Z hydrogels with acceptable adhesion properties and antibacterial properties, enhancing the stability of the implanting environment after bone transplantation. HCA, hydrocaffeic acid; 2-Mclm, 2-methylimidazole; VEGF, vascular endothelial growth factor (Reprinted with permission from Ref. [113]. Copyright 2020 American Chemical Society).

As an essential member of MOFs, zeolitic imidazolate frameworks-8 (ZIF-8) is a monocrystal constructed of Zn^2+^ that connects to each other [110]. Recently, Zhang’s study generated antibacterial ZIF-8 using the diethanolamine template and solvent techniques [111]. The ZIF-8 synthesized in these two techniques exhibits remarkable antibacterial activity and is biocompatible at low concentrations. Taking advantage of its prolonged release of Zn^2+^, which is essential in bone regeneration, revascularization, and antimicrobial activities, ZIF-8 has the promise to be applied as a modification material in bone tissue engineering. When applied to rat bone marrow stromal cells (rBMSCs), ZIF-8 activated the extracellular-signal-regulated kinase (ERK) pathway primarily, and eventually activated the classical mitogen-activated protein kinase (MAPK) signaling and promoted osteogenesis. [112]. For example, Liu et al. designed ZIF-8 nanoparticles (ZIF-8 NPs) functionalized catechol-chitosan (CA-CS) hydrogels (CA-CS/Z) to guarantee adequate blood supply, maintain the stabilization of the bone transplant environment, enhance osteogenesis, and promote bone regeneration (Figure 4) [113]. The hydrogel demonstrated satisfactory adhesion and antimicrobial activities. ZIF-8 discharged from hydrogels may also increase the release and formation of osteocalcin, collagen I, and ALP, hence enhancing rBMSCs’ osteogenic differentiation.

Nonetheless, excessive metal ions produced by MOFs may be toxic to human cells [51,114]. Numerous institutions are researching ways to improve the stability of metal ions as a solution to this issue. Zheng et al. fabricated a nanoplate with a gallic-acid-magnesium-based MOFs (Mg-MOF) core and a biodegradable calcium phosphate (CaP) shell [115]. With the shell in place, the core was less susceptible to degradation, and the bioactive components contained within were more likely to reach a prolonged release under low-pH conditions stimulated by cytokine interleukin-4 (IL4). Then, IL4-MOF@CaP was integrated into collagen (Col) to create a biodegradable scaffold with significant bone regeneration. In addition to being composed of metal ions with antibacterial properties to exert antibacterial effects, MOFs can be loaded with various antibacterial agents as carriers [116]. For instance, Huang et al. successfully constructed an intelligent and long-lasting agent carrier of MOFs(HKUST-1)@carboxymethyl chitosan (HKUST-1@CMCS) [117]. These results indicated that dimethyl fumarate-loaded carrier had enhanced and long-lasting antibacterial action.

### 3.3. Light-Mediated Organic Antibacterial Hydrogels

Organic photothermal agents are categorized into two types: organic nanoparticles (such as porphyrin–lipid conjugate porphysome and organic semiconducting polymer nanoparticles) and organic dye molecules (such as indocyanine green (ICG), IR820, IR780) [70,118,119]. These photothermal conversion materials are biodegradable but easily photodegradable or photobleached [120].

Kuang et al. developed an injectable multifunctional hydrogel for NIR-triggered release for bone regeneration. This hydrogel consisted of poly (dimethylaminoethyl methacrylate-co-2-hydroxyethyl methacrylate)-coordinated situ-generated CaP nanoparticle (ICPN) (poly (DMAEMA-co-HEMA)/ICPN) (DHCP) hydrogel loaded with poly (N-acryloyl glycinamide-co-acrylamide) (PNAm)-ICG- parathyroid hormone (PTH) microspheres (PIP MSs) [121]. Through the photothermal activity of ICG and the thermal polymerization of PNAm, the temperature was rapidly raised, so that PTH can be released accurately and controlled. The injectable NIR (808nm)-light-responsive hydrogel may stimulate osteoblast and osteoclast activity simultaneously and repair cranial defects successfully.

Additionally, served as PTAs, Polydopamine (PDA) exhibits excellent photothermal conversion and adhesion abilities [121,122]. Luo et al. combined immobilized cisplatin with PDA-modified nano-hydroxyapatite (HA) in an injectable hydrogel composed of oxidized sodium alginate and CS. In animals, the hydrogel had photothermal anticancer effects and facilitated the growth of new bone structures [123]. Yao et al. prepared HA, PDA, and carboxymethyl chitosan (CMCS) composite scaffolds [124]. In vitro, the scaffolds with PDA may stimulate higher BMSCs’ osteogenic differentiation than scaffolds lacking PDA. Additionally, the effect of the photothermal process on the osteogenic differentiation was not affected.

The disadvantage of organic photothermic agents is their susceptibility to photobleaching. Not only are conventional organic NIR-absorbing compounds difficult to synthesize, but they are also prone to photobleaching when exposed to light. These disadvantages result in increased costs and the possibility of performance degradation in PTT. Organic photothermal agents must therefore be modified or packaged to maintain their photothermal capabilities [125].

## 4. Hybrid Hydrogels with Natural Antibacterial Agents for Bone Defect Repair

Natural antibacterial agents can be classified according to their sources, including microorganism origin (antibiotics such as vancomycin [126], Aspergillomarasmine A [127]), plant origin (curcumin (Cur) [128], quercetin [91]), and animal origin (antimicrobial peptides (AMPs) [129]). As a matter of fact, the majority of antibiotics currently used or under investigation are produced from secondary metabolites extracted from microbial pathogens, including gentamicin, penicillin, erythromycin, and chloramphenicol [130]. Plant extracts are diverse in composition because even from the same plant, numerous extracts with varying compositions can be prepared by altering the extraction conditions. Due to the inherent activity of natural antibiotics, the extracts of lysozymes, AMPs, and antimicrobial proteins from natural substances are a crucial focus of animal origin antimicrobial agent development [131]. AMPs, which are also called host defense peptides (HDPs), are found in all living animals. They are essential parts of the innate immune system’s response to pathogens [132,133]. In vivo, AMPs have the primary biological function of eliminating harmful microbes such as GPB and GNB, fungi, and viruses [134]. Aside from their antibacterial effect, it has also been shown that AMPs are essential in intracellular processes such as angiogenesis, inflammation, and cell signaling, making them potential candidates for creating new medications [135].

### 4.1. Hybrid Hydrogels with Microorganism Origin Natural Antibacterial Agents

Antibiotics are antibacterial organic compounds derived from natural microorganisms or synthesized in the laboratory. Antibiotics are the most frequently prescribed treatments in hospitals and clinics for bacterial illnesses. Both in therapy and prevention, they are frequently employed in clinical care. Antibiotics have a wide range of antibacterial mechanisms at their disposal. Aside from affecting cell walls and proteins, they can also harm DNA replication and disrupt metabolic processes [136]. Traditionally, broad-spectrum antibiotics are applied systemically to treat bone infections. Antibiotics such as gentamicin and vancomycin are commonly utilized in clinic to treat IBDs [137,138].

Internal encapsulation/physical entrapment through the hydrogels is a strategy for achieving prolonged, localized antibiotic release, hence minimizing systemic adverse effects of antibiotic treatment [139]. This is particularly critical for managing osteomyelitis, which often requires prolonged courses of antibiotics at high doses. Some antibiotics affect osteogenic activities in vitro. According to recent research, a low dose of doxycycline can promote osteogenic differentiation during the initial stages of the procedure [140]. Park JB. et al. showed that increasing tetracycline levels could result in a dose-dependent inhibition in osteogenesis and cell differentiation [141]. A co-delivery system can be built to deliver antibacterial and osteoinductive medicines concurrently or sequentially. Jung et al. fabricated an alginates (ALG)/hyaluronic acid (HA) hydrogel that gelled in situ and comprised BMP-2 and vancomycin [142]. The hydrogel successfully inhibited bacteria proliferation of osteomyelitis and promoted bone repair without the use of supplemental bone transplants. Additionally, the femur treated with the hydrogel regenerated bone more densely compared to the other groups. Only checking the influence of antibiotics on osteogenic activities is insufficient for antibiotics with osteogenic and antibacterial capabilities. The impact of their different concentrations on osteogenesis activity should also be investigated. Liu et al. composited calcium phosphate bone cement (CPC) with gelatin–alginate hydrogels impregnated with gentamicin (GS) in various ratios of 0, 12.5, 25, and 50 vol% [143]. As a result of the findings, the C/0.5-GS complex had the most excellent antibacterial effect and was non-cytotoxic. However, it decreased cell mineralization. The result indicated that high levels of GS in CPC inhibited the capacity of ALP. As a result, C/0.25-GS could be chosen as the best composite due to its adequate strength, steady and sustainable antibiotic release ability, antibacterial activity, and bio-reactivity. An ideal balance between growth factor and drug is necessary for bone formation because high antibiotic doses may hinder osteoblastic differentiation [144].

Antibiotic-resistant bacteria have been increasingly prevalent during the last few decades [145]. Antibiotic therapy is frequently ineffective in osteomyelitis as a result of impaired local vasculature [146]. Furthermore, antibiotics have been proven to be harmful to mammalian cells, resulting in mitochondrial malfunction [147]. The high occurrence of severe bone infections and the increasing risk that antibiotics may become less effective necessitates the development of non-antibiotic-based treatments to replace antibiotics.

### 4.2. Hybrid Hydrogels with Plant Origin Natural Antibacterial Agents

As a result of the excellent biocompatibility and biodegradability, natural antibacterial agents are the first antibacterial agents utilized by humans. They are derived from certain animals and plants with antibacterial activity [90].

Curcumin is a polyphenolic organic molecule derived from turmeric [148]. A series of studies revealed that Cur had antibacterial and anti-inflammation activities [128,149], enhanced osteoblasts’ proliferation, and induced osteogenesis-related gene expressions [150,151]. Various investigations have demonstrated that curcumin possesses broad-spectrum antibacterial properties as well as significant biological activity against both GPB and GNB [152]. The antimicrobial mechanistic methods of curcumin typically entail interfering with cellular division as well as the stimulation of the temperature-sensitive protein-filamenting mutant Z. (FtsZ) [153]. The FtsZ protein is related to cell replication in microorganisms, and it is the first protein to appear at sites about to divide [154]. Curcumin is a photosensitizer with phototoxicity that has been shown to have bactericidal effects on various bacteria when exposed to blue light [155,156,157]. Moreover, investigations have demonstrated that methoxy and hydroxyl of Cur are directly related to its antibacterial properties [158]. Unfortunately, it is challenging to combine hydrophobic curcumin with hydrophilic hydrogels. The low solubility and bioavailability restrict the use of curcumin in clinic. So far, many efforts have been made to encapsulate curcumin. Through the use of photocuring and ethanol treatment, Yu et al. were able to develop Cur-loaded CS nanoparticles (CCNP) in SF/hyaluronic acid esterified by methacrylate (HAMA) hydrogel (CCNPs-SF/HAMA) [159]. In vitro study revealed that the hydrogel showed anti-cancer properties while also enhancing osteoblast growth when the concentration of Cur was 150 g/mL. Virk et al. used an electrophoretic deposition technique to create a multilayer coating containing CS and Cur to give orthopedic implants biological and antibacterial abilities. Both characteristics indicate the prospects of the novel material for bone regeneration [160].

Similar to curcumin derived from plants, cannabidiol (CBD) is an ingredient obtained from the Cannabis sativa with anti-inflammatory, antibacterial activity, and the ability of regulating bone metabolism [161,162,163]. CBD has also been found to enhance the migration of MSCs by activating the P42/44 MAPK signaling pathway and subsequently differentiating into osteoblasts [164]. Qi et al. developed a Cu-alginate hydrogel containing CBD (SA@Cu/CBD) for bone regeneration [165]. The hydrogel was antimicrobial and suppressed the inflammatory response while also promoting osteoblast differentiation and exhibiting angiogenic properties.

### 4.3. Hybrid Hydrogels with Animal Origin Natural Antibacterial Agents

AMPs have broad-spectrum antibacterial activity by cationic and hydrophobic residues [166,167]. Various mammalian cells synthesize AMPs such as defensins, cathelicidins, and histatins [168]. The antimicrobial properties of AMPs were widely believed to be based on their capacity to disrupt membranes via the amphipathic scaffold [169]. AMPs derived from small amino acids would rarely deposit in the human body and could be promptly eliminated from the body [170].

Previous studies demonstrated that AMPs have negligible induction of bacterial resistance. Thus, they can be used to limit microbial contamination in biomedical implants by delivering locally [171]. AMPs cooperated with an appropriate scaffold material to promote bone repair is one of the effective methods in the treatment of IBDs. Yang et al. synthesized a self-assembling hydrogel that RADA16 loaded with AMPs, and the RADA16-AMP had a significant impact on bone growth [172]. Cheng et al. formed a gelatin-based hydrogel containing catechol motifs [173]. Additionally, then, the hydrogel composition was backed with a short cationic antimicrobial peptide (HHC-36) and synthetic silicate nanoparticles (SNs). The hydrogel showed unique features, including strong adhesion, antibacterial activity, and promoting osteogenesis. Sani et al. reported a hydrogel made of gelatin and AMPs that was triggered by visible light [174]. The GelAMP demonstrated excellent antibacterial properties against Porphyromonas gingivalis and promoted bone regeneration in mice.

Some antimicrobial peptides also have an effect on osteogenesis. Due to its broad-spectrum antibacterial activity and multiple bio-functions, particularly osteogenic stimulation, antimicrobial peptides LL37 are regarded as a promising option for bone tissue engineering [175]. LL37 can enhance proliferation, migration, and osteogenic differentiation of MSCs and block bone resorption [176]. Liu et al. fabricated a scaffold for subchondral bone regeneration utilizing LL37-modified layered double hydroxide/CS (LL37@LC) [177]. The study demonstrated that the scaffold might differentiate MSCs into osteoblasts and promote vasculogenesis. Although natural antibacterial agents have a wide range of sources and excellent biodegradable ability, they do have some drawbacks, including insufficient antimicrobial activities or unstable antimicrobial activities.

## 5. Hydrogels with the Inherent Antibacterial Ability for Bone Defect Repair

Besides the antimicrobial agents, the carrier materials (hydrogels) also have antibacterial activity. CS is a natural biopolymer that resembles hyaluronic acid in structure, which has the inherent antibacterial ability and can disrupt cytomembrane structure, cellular energy metabolism, and protein synthesis [174,178,179]. According to the findings of this study, CS promoted the expression of calcium-binding and mineralization genes, including osteocalcin, osteonectin, osteopontin, and collagen type I alpha 1 (COL1A1) [180]. Typically, CS is frequently mixed with osteogenic agents to form hybrid composites suitable for orthopedic biomedical implants, such as RGD ligand [181]. RGD-modified CS decreased the adhesion of S. epidermidis and S. aureus by 85% and 67%, respectively. Additionally, it promoted the expression of osteogenic markers. Hydroxypropyltrimethyl ammonium chloride chitosan (HACC), a new water-soluble CS derivative, has a broad-spectrum antibacterial activity and has been effectively utilized in bone regeneration as an antibacterial agent. Wang et al. developed the HACC/BMP2-BioCaP complex, which was capable of quickly releasing HACC, accompanied by a sustained release of BMP-2 in critical-sized IBDs [12]. Huang et al. used a photo-crosslinking approach to incorporate hydroxyapatite (HAp)@PDA-F nanoparticles with the quaternized and methacrylated CS (CS/HAp@PDA-F) [182]. The hydrogel system preserved osteogenic differentiation potency and provided an excellent antibacterial activity.

**Figure 5:**
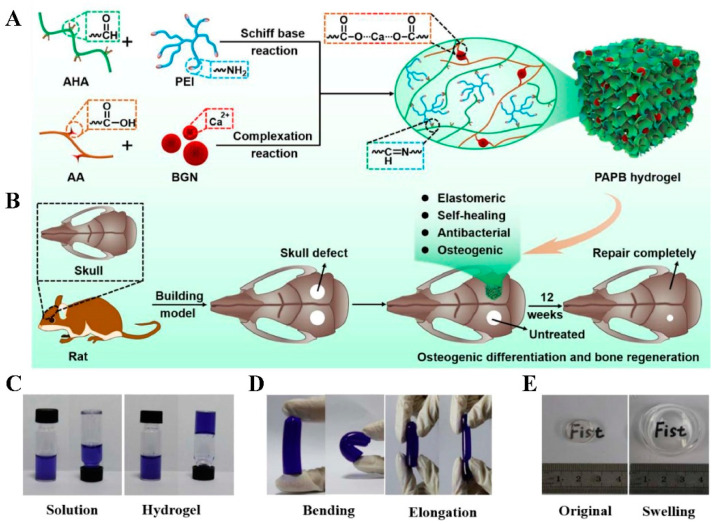
Schematic illustration showing the synthesis process of multifunctional PAPB hydrogel and the effective application. (**A**) The synthesis process of multifunctional PAPB hydrogel, (**B**) potential biomedical applications of multifunctional PAPB hydrogel; (**C**) Intuitive optical images of before and after gelation; (**D**) Intuitive optical images of bending and elongation; (**E**) Intuitive optical images of before and after swelling. AA, acrylic acid (Reprinted with permission from Ref. [187] Copyright 2022 Elsevier).

Some chitosan-based composites have been modified to improve their mechanical qualities and antibacterial activity, such as grafting PEI onto chitosan, grafting chitosan onto PEI, or creating a chitosan-PEI composite [183]. PEI includes a 1:2:1 ratio of primary, secondary, and tertiary amino groups. It is known that PEI can improve the bactericidal efficacy of both hydrophilic and hydrophobic antibacterial agents, and it is also a frequently used microbicidal component in its own right in microbiology [184]. They possess permeabilizing properties and are capable of damaging the membranes of bacteria [185,186]. Li et al. reported a self-healing bioactive antibacterial nanocomposite hydrogel based on crosslinking poly polyacrylate/aldehyde-hyaluronic acid (AHA)/PEI/bioactive glass nanoparticles (BGN) (PAPB) in a triple-network configuration. The hydrogel showed favorable biomineralization activity, which facilitated the reconstitution of skull defects (Figure 5) [187].

## 6. Summary and Challenges

To prevent the bone substitutes from being infected during repair, osteoconductive scaffolds that maintain the release of antibacterial agents over the 4 to 6 week duration for complete vascularization are necessary [188]. As a result, there is an immediate requirement for the development of bone-implant materials that provide long-lasting antibacterial activity and stimulate bone repair [189]. The present review summarizes the current development of the hybrid hydrogel with inorganic, organic, and natural antibacterial agents. Table 1 summarizes the advantages and disadvantages of different antibacterial agent hybrid hydrogels. Although adding antibiotics to hydrogel can enhance the antibacterial properties of materials and increase the speed of bone repair, insufficient long-lasting antimicrobial capability and insufficient osteogenesis properties result in unsatisfactory tissue regeneration [189]. Antibiotics and antibacterial metals, such as Ag, Cu, and Au, have already been implemented into hydrogels to treat and prevent bone infection. However, the risk of antibiotic resistance and tissue toxicity from metal ion release may limit their clinical use [190,191]. Light-mediated antibacterial agents offer a solution to the problem of bacterial resistance and tissue toxicity through their unique antibacterial mechanism.

Recent research indicates that PTT or PDT can promote the proliferation of cells and osteogenesis differentiation, and some nanomaterials possess intrinsic or light-triggered bactericidal properties. Furthermore, the photothermal treatment kills microorganisms by raising the local temperature, causing physical damage to bacteria, and preventing the development of antibiotic resistance. Although light-mediated antibacterial mechanisms have been recognized as one of the most effective antibacterial approaches, their ability to target organisms, oxygen-deprivation-infected tissues, as well as photocatalytic efficiency are still significant variables restricting their antimicrobial effectiveness [192]. To satisfy the future requirements of light-mediated antibacterial agents, it is expected to develop innovative light-mediated antibacterial agents with adequate size, excellent photostability, high photothermal conversion efficiency, and low toxicity for effective PTT and PDT for infection treatment and bone repair. Furthermore, in comparison to PTT or PDT alone, the combination treatment exhibited a synergistic effect, leading to increased efficacy of treatment without noticeable toxic consequences on normal tissues [193]. Therefore, combined PTT and PDT hold desired promise for the treatment of IBDs.

It is to be regretted that the cell and animal investigations of antibacterial agents hybrid hydrogels mentioned above have not yet been applied in clinic. To date, no investigations have described the use of antibacterial hybrid hydrogels for the clinical treatment of IBDs. It is difficult to directly apply the results of successful in-human cell or animal studies to clinical experience. As a result, clinical trials evaluating the safety and functional effectiveness of hybrid hydrogels with antibacterial agents are required in the future. Additionally, a promising future direction is the use of multifunctional materials paired with systemic and local therapy for the treatment of IBDs, and different methods of treatment should be used wherever possible, including multiple drugs, co-delivery, and hyperthermia [194].

In conclusion, antibacterial agents such as antibiotics, metal particles, and AMPs are usually incorporated into hydrogels to endow them with antibacterial activity. For some hydrogels with inherent antibacterial capability, it is convenient to adjust the biocompatibility and antibacterial activity of the hydrogels via chemical modification in various ways. The promising way to treat IBDs is to create a bone graft with antimicrobial and osteogenesis properties in sequential order. Despite significant progress, hydrogels possessing the activities of anti-inflammatory, antibacterial, osteogenic, and angiogenic are desperately needed to treat IBDs.

**Table 1:** Summary of different antibacterial agents hybrid hydrogels for infected bone repair.

Category	Representative Agent	Antibacterial Mechanism	Effect on Bone Repair	Advantages	Disadvantages	Ref.
Hydrogels with metal nanomaterials	AgNPs	Attach onto the cell wall and membrane, damage intracellular biomolecules and structures	Promote the expression and mineralization of osteogenic proteins, alter microRNA expression associated with bone formation	Broad-spectrum antimicrobial properties, stimulate bone growth	Long-term use produces multidrug-resistant bacteria and is difficult to biodegrade	[51,195]
Light-mediated inorganic antibacterial nanoparticle hybrid hydrogels	rGO	Mechanical breakage of the cell membrane results in intracellular substance leakage	Promote cell proliferation and differentiation	Do not elicit bacterial resistance	Low photothermal conversion efficiency, non-biodegradable nature	[196,197]
Hydrogels with organic antibacterial agent	Quaternary ammonium salts	Binding to the cell membrane, bacteria lysis	Promote more osteogenic differentiation	Can be used as a modification factor	Short-term functionality, environmental toxicity, rapid antimicrobial resistance, and skin penetration	[96,97,198]
Hydrogel with MOFs	ZIF-8	Synergistic action, such as Zn^2+^ and ligand release, ROS production, photothermal effect	Activate the ERK pathway primarily, activates MAPK signaling eventually, and promotes the osteogenesis of rBMSCs	Can be used as carriers and have electrostatic interaction with negatively charged bacterial cells	Excess metal ions may be harmful to host tissues	[112,199]
Light-mediated organic antibacterial agent hybrid hydrogels	ICG	Combination of PTT and PDT to kill bacteria through ROS generation and thermal ablation	Increase ALP activity and enhanced mineralization of osteoblasts	Water-soluble, very low cytotoxicity	Rapid clearance from the body, instability in aqueous solutions, an photobleaching	[200,201,202,203,204,205]
Hydrogels with microorganisms origin natural antibacterial agents	Doxycycline	Interfere with prokaryotic protein synthesis at the ribosome level	Promote by low concentration, but inhibit by high concentration	Broad-spectrum antibacterial drug	Antibiotic-resistant bacteria, toxic to mammalian cells	[140,141,206]
Hydrogels with plant origin natural antibacterial agents	Cur	Target the bacterial DNA, protein, cell membrane, cell wall, and other biological components	Enhance osteoblast proliferation, and induce osteogenesis-related gene expression	Wide sources and good biodegradability	Poor solubility and bioavailability	[149,150,151,155]
Hydrogels with animal origin natural antibacterial agents	LL37	Induce membrane rupture	Enhance proliferation, migration, and osteogenic differentiation of MSCs and block bone resorption	Broad-spectrum activity against	Insufficient antimicrobial activities or unstable antimicrobial activities	[176,207,208]
Hydrogels with inherent self-antibacterial ability	CS	Disrupt cytomembrane structure, cellular energy metabolism, and protein synthesis	Up-regulate genes associated with calcium binding and mineralization	Environmentally friendly agent and cytocompatibility	Limited bacterial activity against Gram-negative bacteria	[209,210]

## Data Availability

Not applicable.
